# Phase I trial of oral etoposide in combination with radiotherapy in head and neck squamous cell carcinoma - GORTEC 2004–02

**DOI:** 10.1186/1748-717X-8-40

**Published:** 2013-02-27

**Authors:** Yungan Tao, Etienne Bardet, Dominique Rosine, Frédéric Rolland, Emmanuelle Bompas, Nicolas Daly-Schveitzer, Antoine Lusinchi, Jean Bourhis

**Affiliations:** 1 Institute Gustave-Roussy, Villejuif, France; 2 Centre René Gauducheau CLCC, Nantes, France

**Keywords:** Oral etoposide, Head and neck cancer, Radiotherapy, Chemotherapy, Phase I

## Abstract

**Purpose:**

This study sought to determine the maximum tolerated dose (MTD) of oral etoposide in combination with radiotherapy in head and neck squamous cell carcinoma (HNSCC).

**Patients and Methods:**

Phase I, multicenter, open-labelled, non-comparative and dose escalating trial. Patients with locally advanced HNSCC were enrolled onto cohorts of escalating dose of etoposide. Oral etoposide was administered on five consecutive days every week for 7 weeks (7 treatment cycles) in combination with daily radiotherapy (70 Gy /35 fractions). Two dose levels (25 mg/day and 50 mg/day) of etoposide were planned and three to six patients were to be enrolled at each level according to the potential DLTs.

**Results:**

Fourteen patients were allocated to two dose levels: 25 mg/day (3) and 50 mg/day (11). Cisplatin was contra-indicated in all the patients included. Only one patient (50 mg/day) presents a grade 4 neutropenia (DLT), no other DLTs were observed. The most frequently adverse events (AEs) were radiomucositis. Two deaths before 3 months of end of treatment were not related to treatment. Seven patients were still alive with a median follow-up of 30 months (12–58 months). Nine patients had a complete response (CR) at 3 months after the radiotherapy; Among the 9 patients, 3 patients had a local relapse; one patient with local and distant relapse.

**Conclusion:**

Due to only one DLT experienced, it is possible to a dose of 50 mg/day for phase II studies, however this should be considered with caution.

## Background

Treatment of inoperable locally advanced head and neck squamous cell carcinomas (HNSCC) is based on radiotherapy (RT) delivering 70 Gy/ 7 weeks. Chemotherapy is an integral part of the treatment of locally advanced HNSCC, while added to radiotherapy [1]. Concurrent chemo-radiotherapy (CRT) has become a standard treatment of locally advanced HNSCC, as reported in the Meta-Analysis of Chemotherapy in Head & Neck Cancer (MACH-NC). A recent update has demonstrated an absolute survival benefit of 6.5% at 5 years in favour of adding concurrent chemotherapy to RT [2]. The gain of survival reaches 11% at 5 years when chemotherapy is cisplatin alone in combination with RT. In a randomized phase III trial GORTEC 94–01, we showed the superiority of concurrent CRT (carboplatin and 5FU with RT 70 Gy/ 7 weeks) compared with RT alone in tumor control. However, these improvements in tumor control were accompanied by an increase of acute toxicity (hematologic, mucositis etc.), and also of late toxicity [3]. It is therefore essential to optimize these associations of CRT for more effective and less toxic effects.

The rationale for the chemo-radiotherapeutic combinations relies on spatial cooperation or interaction between modalities [4]. Interactions may take place at the molecular level, with altered DNA repair or modification of the lesions induced by drugs or radiation; at the cellular level, notably through cytokinetic cooperation arising from differential sensitivity of the various compartments of the cell cycle to the drug or radiation; and at the tissue level, including reoxygenation, increased drug uptake or inhibition of repopulation or angiogenesis [4]. Many chemotherapeutic agents have demonstrated a radiosensitizing effect [5,6]. Among the drugs potentially interesting in the context of these optimizations, etoposide (VP-16, Celltop®) is of particular interest. The topoisomerase inhibitor etoposide is a semisynthetic derivate of epipodophyllotoxin which has proven activity in several malignant diseases and is commonly used for treating germ cell tumors, haematological malignancies, and small cell lung cancer [7]. Etoposide has been found to act as a radiosensitizer and is able to potentiate the effect of irradiation when used in experimental models [6]. Moreover, it is possible to use etoposide orally, thus limiting practical difficulties related to the administration of drugs (no hospitalization, hydration etc.). However, the tolerability of combination of concurrent oral etoposide and radiotherapy are poorly understood.

The latest pharmacokinetic data showed that the clinical effects observed with oral etoposide vary depending on the dose and especially the splitting of the dose. Thus the toxicity was much reduced by administration of 75 mg divided in 3 doses of 25 mg, compared with a single dose of 75 mg which was responsible for a high serum peak and significantly higher toxicity. When used in association with radiotherapy, it is possible that the divided dose of etoposide is a factor in determining the tolerance of combined treatment.

The aim of the study was to evaluate the feasibility and dose-limiting toxicity (DLT) of the combination of radiotherapy and oral administration of etoposide in patients with locally advanced HNSCC. The principal objective was to evaluate the DLT and to determinate the level of maximum tolerated dose (MTD) and the secondary objective was to evaluate the overall survival and loco-regional control rate.

## Methods

### Study design

This open-label, non-comparative, dose-escalation study was conducted in two centers in France with approval of Kremlin-Bicêtre Ethics Committee (France). Seven cycles of Celltop® were administered on five consecutive days/ week, in conjunction with continuous conventional radiotherapy. Two dose levels (25 mg/day and 50 mg/day) of Celltop® were planned in order to recruit patients according to the potential DLTs experienced. Etoposide was delivered orally in two groups: 25 mg/day (1 capsule of Celltop® 25 mg administrated orally 2 hours before daily irradiation); 50 mg/day (2 capsules of Celltop® 25 mg or 1 capsule of Celltop® 50 mg administrated orally).

Dose escalation was considered when the last patients receiving the inferior dose level had completed the treatment plus 2 weeks follow-up. Patients were initially enrolled at the first dosing level of 25 mg/day and the second dosing group of 50 mg/day was further planned providing the maximum tolerated dose (MTD) was not reached. At least three patients were included in each dose level. Capsules containing 25 mg or 50 mg of Celltop® were administered to patients and taken orally daily over five consecutive days, every week for seven weeks (seven cycles). Assignment of patients to each dose level was centralized (2 centres participating) in order to ensure that an adequate number of patients had completed the necessary dose level and the assessment of toxicity before escalating to the next dose level. Three to six patients were to be enrolled at each dose level according to the occurrence of DLTs. At the second dose level, in the absence of DLTs, additional 5 patients were to be enrolled to confirm that the MTD was reached.

Definition of the occurrence of DLTs: All Grade 3–4 toxicities (NCI Common Toxicity Criteria, version 3) were considered as DLTs, except for: grade 3–4 mucositis toxicity was expected during combined radiation-chemotherapy; grade 3 dermatitis was not considered as DLT; hematologic toxicity (grade 3 thrombocytopenia / non-febrile grade 3 neutropenia). Celltop® dose escalation was determined by the number of patients experiencing Dose Limiting Toxicities (DLTs) in order to elucidate the MTD. A MTD was defined as the dose of Celltop® in combination with radiotherapy in which more than 1 of 6 patients presented a DLT.

The laboratory tests (haematology and biochemistry) were performed at screening and once a week during the treatment period and at each visit of the follow-up period, similarly, performance status was also assessed at every time-point.

### Patients

Before being enrolled in the study, written informed consent was obtained from each patient. The main eligibility criteria were: T1-T4 N0-N3 corresponding to stage III or IV (UICC 2002) or T0 only if N2-N3, oropharynx, hypopharynx, larynx or oral cavity non-operated histologically proved squamous cell carcinoma; Age more than 20 years; Karnofsky performance status > = 70; not amenable to a combined radiation and surgery because of the loco-regional extension and / or general medical state; Laboratory tests compatible with the chemotherapy (platelet count >150 000/μl, neutrophiles > 2000/μl, creatinine serum level < 130 μmol/l and SGOT, SGPT, alkaline phosphatase, bilirubin less than two and a half times normal). All patients were required to sign the informed consent document that was approved by each Institutional Review Board before initiating protocol therapy. A patient was considered unsuitable for inclusion in case of: previous RT or chemotherapy; infection; bulky lymph node (> 6 cm, N3) is not recommended, but will be left to the discretion of each investigator.

The initial assessment for inclusion in this trial include: a general clinical examination; a local and regional tumour staging; an assessment of tumor spread and second location research (head and neck, bronchus, oesophagus) by a pan endoscopic examination under general anesthesia; CT and / or MRI; chest X-ray and liver ultrasound; according to the clinical context, bone scan and / or PET Scan; other complementary examinations; ECG as complete heart work-up if necessary; blood cells count, electrolyte, creatinine, calcium, glucose, alkaline phosphatase, bilirubin, transaminases, prothrombin.

### Radiotherapy

In conjunction with etoposide, patients received concomitant radiotherapy 2 Gy/fraction, 5 fractions per week, up to 70 Gy in 35 fractions and seven weeks. Positive lymph nodes received 64 to 70 Gy and prophylactic neck lymph node levels were delivered to 50 Gy. Radiation was delivered with 6 MV photon beams, using a conventional treatment planning system or three-dimensional (3D) conformal radiotherapy. Reproducible immobilization was required. 3D treatment planning using CT-based tumor and normal tissue definition was recommended.

### Evaluation of response

Evaluation of response was carried out at 3 months after the end of radiation and included physical examination, CT or MRI of the head and neck and / or PET/CT. Complete response was defined as a complete disappearance of tumor. Partial response was defined as a decrease of 50% or more in the sum of the products of diameters of all measurable lesions. Patients were followed every 3 months with clinical examination and CT or MRI of the head and neck and / or PET/CT in case of suspicion of relapse.

## Results

### Patient characteristics

Two dose levels (25 mg/day and 50 mg/day) of Celltop® were used in this trial as initially planned. Three patients were enrolled at the dose level of 25 mg/day. Three patients were initially enrolled at the dose level 50 mg/day, and 3 further patients were enrolled because of the occurrence of one DLT, and then 5 additional patients were enrolled in the second level for a confirmation of MTD. As shown in Table 1, 14 patients were included and the majority of patients were male (13/14) with a median age of 58 years (51–78 years), and 7/14 patients with chronic cardiac disease (2 with myocardial infarctus, 3 with cardiomyopathy, 3 with heart failure), 4/14 patients with pulmonary associated disease (2 with chronic obstructive pulmonary disease, 1 with pulmonary embolism and 1 with respiratory failure). Most patients were heavily tobacco-toxic and there is also 1 patient with hypoacusia and 2 other patients with stroke and diabetes. Cisplatin was contra-indicated in all the patients included. The most frequent tumor site was oropharynx (7 patients), and 2 patients with laryngeal cancer, 3 with hypopharynx and 2 with oral cavity. All the patients had a biopsy proven squamous cell carcinoma. All had tumors staged as T2 to T4, N0 to N2c and M0 according to UICC 2002 classification (Table 2).

**Table 1:** Patient demographics

***Characteristics***	***Number of patients (%), n=14***
*Gender*	
*Male*	*13 (93)*
*female*	*1 (7)*
*Age (years)*	
*Median*	*58*
*Range*	*51-78*
*Comorbidities*	
*Chronic cardiac disease*	*7 (50)*
*Pulmonary disease*	*4 (28)*
*UICC stage*	
*III*	*6 (43)*
*IV*	*8 (56)*
*Primary site*	
*Oropharynx*	*7 (50)*
*Hypopharynx*	*3 (20)*
*Larynx*	*2 (15)*
*Oral cavity*	*2 (15)*

**Table 2:** TNM staging

	***T1***	***T2***	***T3***	***T4***	**Total**
**N0**	*0*	*0*	*1*	*3*	*4*
**N1**	*1*	*1*	*1*	*1*	*4*
**N2a**	*0*	*0*	*1*	*0*	*1*
**N2b**	*2*	*1*	*0*	*0*	*3*
**N2c**	*0*	*0*	*0*	*0*	*0*
**N3**	*0*	*0*	*2*	*0*	*2*
**Total**	*3*	*2*	*5*	*4*	*14*

### Treatment

#### Radiotherapy

All the included patients completed the planed irradiation 68–70 Gy except two patients, one at the dose level of 50 mg/day, stopped at 42.5 Gy and then died of pulmonary emboli which was considered as not related with the RT-CT; another patient, also at the dose level of 50 mg/day, stopped at 10 Gy and died of cardiac disease. Among the patients who completed the whole course radiotherapy, in one patient at the dose level of 50 mg/day, the radiotherapy had been interrupted more than one week (11 days) because of febrile disease (there is no grade 3 neutropenia, however we did not find the exact reason, it is probably because of mucositis, and the interruption of 11 days is not a continuous interruption and it is rather a mixed interruption with also machine maintenance and breakdown); in another patient at the dose level of 50 mg/day, the radiotherapy had been interrupted more than one week (9 days) because of the breakdown of Linac.

#### Chemotherapy

One patient at the dose level of 50 mg/day completed 4 weeks of Celltop® with radiation and stopped at 42.5 Gy and then died of pulmonary embolism; one patient at the dose level of 50 mg/day had never taken the oral Celltop® because of deglutition problem who died after one week of radiotherapy (10 Gy); one patient at the dose level 50 mg/day completed only 4 weeks Celltop® because of febrile neutropenia (DLT see below), and another patient in the same group completed a little less than 6 weeks of Celltop® because of febrile reason (we did not find the exact reason, probably because of mucositis without grade 3 neutropenia). All other 10 patients completed all the planned 7 cycles of Celltop®.

### Safety results

**Table 3:** Toxicity after Celltop® and radiation

	***25 mg/day***	***50 mg/day***
**Mucositis**		
Grade 1/2		3
Grade 3	3	7
**Radiodermitis**		
Grade 1/2	3	6
Grade 3		4
**Dysphagia**		
Grade 1/2	3	6
Grade 3		4
**Leucopenia**		
Grade 1/2	1	1
Grade 3/4		2
**Neutropenia**		
Grade 1/2		1
Grade 3/4		2
**Thrombopenia**		
Grade 1/2		1
**Anemia**		6
Grade 1/2		
**Renal**		2
Grade 1		

Only one patient experienced DLT (febrile grade 4 neutropenia) at the dose level of 50 mg/day at week 5.

Other severe AEs included grade 3 mucositis in 10 patients (3 patients at the dose level of 25 mg/day, 7 patients at the dose level of 50 mg/day). 4 patients at the dose level of 50 mg/day suffered from grade 3 radiodermitis. Two patients with grade 3/4 leucopenia were observed at the dose level of 50 mg/day. One patient with grade 3 neutropenia was observed at the dose level of 50 mg/day. 4 patients at the dose level of 50 mg/day suffered from grade 3 dysphagia.

Grade 1 or 2 toxicities including grade 2 mucositis in 3 patients at the dose level of 50 mg/day ; 6 patients (2 patients at the dose level of 25 mg/day, 4 patients at the dose level of 50 mg/day) suffered from grade 2 radiodermitis and 3 patients with grade 1 radiodermitis (1 patient at the dose level of 25 mg/day and 2 patients at the dose level of 50 mg/day 7 patients (3 patients at the dose level of 25 mg/day, 4 patients at the dose level of 50 mg/day) suffered from grade 2 dysphagia and 2 patients at the dose level of 50 mg/day with grade 1 dysphagia.

Six patients at the dose level of 50 mg/day had grade 1/ 2 anemia. One patient at the dose level of 50 mg/day had grade 2 leucopenia, grade 1 neutropenia and thrombopenia; one patient at the dose level of 50 mg/day with grade 2 neutropenia; One patient at the dose level of 25 mg/day with grade 1 leucopenia. Two patients at the dose level of 50 mg/day had renal toxicity (creatininemia grade 1). Other toxicities such as hepatic and neurological toxicities have not been observed in the present study (Table 3).

One patient with heavy antecedents (coronary heart disease, hypertension and chronic obstructive pulmonary disease) before CRT, at the dose level of 50 mg/day, stopped at 42.5 Gy and then died of pulmonary embolism, and another patient with a T3N3 laryngeal cancer at the same dose level died of acute respiratory distress syndrome with hemorrhagic event of loco-regional disease progression one month after the chemo-radiotherapy. One patient had never taken Celltop® because of deglutition problem and died after one week of radiotherapy at the dose level of 50 mg/day. All these three deaths had been considered as in association with their intercurrent diseases and not with etoposide and radiation.

### Efficacy

Among the 12 patients who completed the whole course of radiotherapy, one patient died of respiratory depresses one month after the completion of radiotherapy at the dose level of 50 mg/day. This is a patient with T3N3 laryngeal cancer and the cause of death is probably respiratory depresses induced by hemorrhagic event of loco-regional disease progression. Seven patients of the 11 evaluable patients were still alive with a median follow-up of 30 months (12–58 months). Nine patients had a tumoral complete response (CR) at 3 months after the radiotherapy; one patient had a CR in the primary tumour but a partial response (PR) of neck lymph nodes, and then progression in mediastinal lymph nodes, pulmonary and cerebral metastases; another patient had a local progression (P) and presented a second oesophageal cancer (Table 4). Among the 9 patients who had a CR at 3 months after radiotherapy, 3 patients had a local relapse; one patient with a local relapse, bone metastasis and a second oesophageal cancer; no other regional relapse and distant metastasis. The causes of death of the four patients were: three with loco-regional disease relapse (including one patient with hemorrhagic local progression at the end of treatment) and one with metastatic relapse.

**Table 4:** Response to treatment

**Response**	**CR**	**PR**	**S**	**P**
**Number patient (%)**	**9 (82%)**	**1 (9%)**	**0**	**1 (9%)**

## Discussion

The meta-analysis MACH-NC demonstrated that concurrent chemo-radiotherapy allowed a significant survival benefit compared to radiation alone and single agent cisplatin appears to be one of the most efficient concurrent treatment in combination with radiotherapy [2]. However, the compliance to the most commonly used cisplatin regimen (100 mg/m2, every 3 weeks [8]) is relatively poor and the concurrent use of cisplatin and radiotherapy has a relatively high toxicity causing adverse effects such as hemato-, nephro-, neurotoxicity, gastrointestinal (GI) toxicity and severe oral mucositis, which make this treatment suitable only for selected patients. The most frequently reported dose-limiting factors with intravenous cisplatin were renal, haematologic and mucositis toxicities. Bernier et al. [8] showed that the rate of severe grade 3–4 mucositis was 41%, severe leukopenia 16%, and severe vomiting was observed in 11% of patients in a randomized study (EORTC 22931).

The recent published results of 10 years follow-up of UK Head and Neck (UKHAN1) trial including 966 patients with LAHNC confirmed the benefit of concurrent CRT which used either methotrexate alone, or vincristine, bleomycin, methotrexate, and fluorouracil [9]. These results suggest that other chemotherapeutic agents have also shown comparable outcome than cisplatin. Among them, etoposide provides a safe and convenient alternative method of administration and has shown activity in lung cancer, ovarian carcinoma, etc. [10].

Etoposide administered orally has been shown previously to be as effective as intravenous etoposide with similar serum half-life, renal clearance, volume of distribution, and toxicity. Oral etoposide has been used in the treatment of patients with various types of cancer with tolerable side effects. Oral etoposide has been investigated as well in patients with advanced head and neck cancer [11]. The palliative effect of oral etoposide has been assessed in heavily pretreated patients with recurrent and/or metastatic HNSCC and oral etoposid has been shown to be markedly effective and an excellent tolerated therapy in this population of severely disabled patients. Oral etoposide was very well tolerated with alopecia and myelosuppression being the most encountered toxicities [11].

Our present study showed the feasibility of combined oral etoposide (25–50 mg/day for 7 cycles) with concurrent conventional radiotherapy with tolerable side effects and comparable efficacy. All the 3 patients with Celltop® 25 mg/day well tolerated all the course of treatment; and most of the 11 patients who received Celltop® 50 mg/day (only one DLT and another patient with grade III neutropenia). The observed mucositis and radiodermatitis are comparable with other reports and all manageable. We could recommend the dose 50 mg/day of Celltop® for use in the future phase II trial. A recent study for HNSCC with 13–27 days of oral etoposide 50 mg/day combined with hyperfractionated radiotherapy has shown similar findings [12]. In our present study, we observed the same profile of toxicities: mucositis and haematological toxicities, however, the dose of etoposide used in our study is higher (50 mg/day for 35 days) than that study (same daily dose for 27 days). Moreover, many patients in our present study presented chronic cardiac or pulmonary co-morbidities and they were considered as not suitable for platinum-based chemotherapy.

Targeted therapies have been shown interesting radiosensitizing effect in the preclinical and clinical setting [13-15]. EGFR inhibition has been shown its efficiency in the treatment of head and neck cancer these recent years with particularly limited toxicities compared with platinum-base chemotherapy. Bonner, et al. reported recently that treatment of advanced HNSCC with concurrent radiotherapy plus cetuximab improves locoregional control and reduces mortality without increasing the common toxic effects associated with radiotherapy [16,17]. Since then, cetuximab and radiotherapy combination has become an alternative of platinum-base concurrent chemo-radiotherapy. In fact, our current trial has been designed and conducted before the publication of results of Bonner’s trial.

One of the concerns of concurrent cetuximab and radiotherapy is severe skin toxicities which have been shown more and more frequently these last years [18-21]. We should also keep in mind that infusion-related complications occur in about 5% of the patients treated with cetuximab [22]. In addition, cardiac toxicity could also be a potential complication associated with cetuximab, especially in patients with prior cardiac comorbidities. Thus, cetuximab needs to be used with caution in fragile patients especially in patients with prior cardiac disease. In our present study, most of the patients suffered chronic cardiac or pulmonary diseases, which limited the use of conventional chemotherapy and even cetuximab should be used cautiously. We obtained a comparable overall survival even in this patient population. We could conclude that concurrent radiotherapy and oral etoposide 50 mg/day for 7 weeks could be another alternative treatment for patients with locally advanced HNSCC, especially for the patients with chronic cardiac or pulmonary co-morbidities. These recent years, HPV-related HNSCC especially oropharyngeal cancer has been shown better survival compared with HPV-non-related HNSCC [23-25]; thus, one of the questions remain is whether other chemotherapeutic agents could obtain comparable disease control as platinum and less toxicities when associated with radiotherapy in HPV-related HNSCC.

## Conclusions

In conclusion, the recommended dose of oral etoposide for phase II trial in combination with radiotherapy could be 50 mg/day, on five consecutive days per week, during the course of radiotherapy, however, this should be considered with caution due to 2 deaths (not related to treatment) before 3 months of the end of treatment.

## Abbreviations

MTD: Maximum tolerated dose; HNSCC: Head and neck squamous cell carcinoma; CRT: Concurrent chemo-radiotherapy; RT: Radiotherapy; MACH-NC: Meta-Analysis of Chemotherapy in Head & Neck Cancer; GORTEC: Groupe oncologie radiothérapie tête et cou; DLT: Dose-limiting toxicity; CR: Complete response; PR: Partial response; LAHNC: Locally advanced head and neck cancer; EGFR: Epidermal growth factor receptor

## Competing interests

The authors declare that they have no competing interests.

## Authors’ contributions

YT, EB, AL and JB participated in data collection, study design and organization as well as preparation of the manuscript, DR, EB and FR also participated in data collection, study design and revision of the manuscript. NDS revised the manuscript. All authors read and approved the final manuscript.
